# Understanding the factors associated with COVID-19 vaccine hesitancy in Venezuela

**DOI:** 10.1186/s12889-024-18598-4

**Published:** 2024-04-23

**Authors:** Fabián R. Chacón-Labrador, María G. Passantino, Augusto Moncada-Ortega, Atahualpa A. Ávila, Andrea A. Moreno, Nicolle A. Kuffaty-Akkou, Luisana M. Pedroza, Natasha A. Camejo-Ávila, Daniela L. Mendoza-Millán, Carlis M. Rodriguez-Saavedra, María V. Marcano-Rojas, Fernando Hernández-Medina, María E. Grillet, Fhabián S. Carrión-Nessi, David A. Forero-Peña

**Affiliations:** 1 Biomedical Research and Therapeutic Vaccines Institute, Ciudad Bolívar, Venezuela; 2 School of Medicine, Universidad Central de Venezuela, Caracas, Venezuela; 3 School of Psychology, Universidad Católica Andrés Bello, Caracas, Venezuela; 4 Immunogenetics Section, Pathophysiology Laboratory, Centro de Medicina Experimental “Miguel Layrisse”, Instituto Venezolano de Investigaciones Científicas, Altos de Pipe, Venezuela; 5 Vector and Parasite Biology Laboratory, Instituto de Zoología y Ecología Tropical, School of Sciences, Universidad Central de Venezuela, Caracas, Venezuela; 6 Infectious Diseases Department, Hospital Universitario de Caracas, Caracas, Venezuela

**Keywords:** COVID-19, Vaccination, Vaccine hesitancy, Knowledge, Attitude, Practice, Venezuela

## Abstract

**Background:**

Despite nearly a quarter of Venezuelans remaining unvaccinated against coronavirus disease 2019 (COVID-19), the factors contributing to vaccine hesitancy in the country have not been thoroughly investigated.

**Methods:**

A cross-sectional study was conducted from October 15^th^ to 30^th^, 2022, using a knowledge, attitudes, and practices (KAP) survey to identify factors associated with COVID-19 vaccine hesitancy.

**Results:**

The study analyzed data from 1,930 participants from all 24 states of Venezuela. The majority (93.4%) were vaccinated. The mean age was 40 years, predominantly female (67.3%), and held a university degree (70.6%). The mean KAP score was significantly higher among vaccinated individuals compared to unvaccinated ones (7.79 vs. 3.94 points for knowledge, 40 vs. 24 points for attitudes, and 16 vs. 10 points for practices, all *p* < 0.001). Increases in the scores for KAP were associated with increased odds of being vaccinated (84.6%, 25.6%, and 33% respectively for each one-point increase, all *p* < 0.001). Certain demographic factors such as marital status, occupation, religious beliefs, monthly income, and location influence COVID-19 vaccine knowledge. Higher income and certain occupations decrease the odds of low knowledge, while residing in specific states increases it. Attitudes towards the COVID-19 vaccine are influenced by age, health status, vaccination status, and location. Higher income and absence of certain health conditions decrease the odds of negative attitudes. Lastly, age, occupation, monthly income, and location affect vaccine practices. Advanced age and higher income decrease the odds of inappropriate practices, while residing in La Guaira state increases them.

**Conclusion:**

Factors such as age, education level, occupation, monthly income, and location were found to be associated with knowledge and attitudes towards COVID-19 vaccine among the surveyed Venezuelans.

**Supplementary Information:**

The online version contains supplementary material available at 10.1186/s12889-024-18598-4.

## Background

Vaccination is widely recognized as the most effective tool for preventing infectious diseases. However, in Venezuela, a low intent to vaccinate against the coronavirus disease 2019 (COVID-19) was documented even before any vaccine was approved and distributed to the public [1]. The COVID-19 pandemic struck Venezuela during a complex humanitarian crisis that has been ongoing since 2016 [2]. This crisis, rooted in socio-economic instability, has led to shortages of healthcare workers, supplies, and basic services in the Venezuelan health system, and has profoundly weakened the quality of care, compromising primary prevention services, including routine immunization, leading to the re-emergence of vaccine-preventable diseases [3]. If vaccine hesitancy within the community is not addressed, outbreaks of infectious diseases could pose a threat to the country and neighboring regions.

As of November 26, 2023, the proportion of the Venezuelan population that had received at least one dose of a COVID-19 vaccine stood at 78%. This percentage is lower than that of other countries in the region, such as Ecuador (84%), Colombia (85%), Brazil (87%), and Argentina (91%) [4]. Furthermore, the proportion of the population that had completed the primary COVID-19 vaccine series was among the lowest in the region, at 50%, which is below the global average of 64.2%, according to the World Health Organization (WHO) [4]. When vaccines began to be distributed in the region, the reasons cited for COVID-19 vaccine hesitancy included lack of knowledge about their effectiveness and safety, myths about vaccination, individual contraindications such as fear of needles, and structural barriers such as distance to clinics and distrust of the government [5]. The association between demographic, economic, social, and cultural factors and resistance to COVID-19 vaccine needs further study in Venezuela [1]. This will help develop strategies to bridge these gaps locally. Therefore, this study aims to identify factors related to knowledge gaps, negative attitudes, and inappropriate practices among both vaccinated and unvaccinated individuals against COVID-19 in the Venezuelan population. The study employs a knowledge, attitudes, and practices (KAP) methodological approach to achieve this goal.

## Methods

### Study design

An online cross-sectional survey was conducted in Venezuela from October 15^th^ to 30^th^, 2022, using the “Google Forms” platform (Google LLC, Mountain View, CA, USA). The study population included participants aged 18 and over, irrespective of their COVID-19 vaccination status. Responses that were inconsistent or incomplete were excluded from the analysis. The survey link was disseminated to potential participants via WhatsApp instant messages and emails. Additionally, the survey link was publicized through flyers posted on primary social media platforms such as Instagram and Twitter (now X). These platforms were affiliated with various national health institutions, including the Venezuelan Society of Infectious Diseases and the Venezuelan Society of Internal Medicine. Participation in the survey was voluntary. To ensure the privacy of the participants, all responses were anonymized and treated with strict confidentiality.

### Sample size

According to the 2022 National Survey of Living Conditions (ENCOVI, in Spanish) in Venezuela [6], the country’s population is approximately 28.3 million. Given a life expectancy of 76 years and assuming a relatively normal age distribution, it may be inferred that about 75% of the population, or 22.9 million individuals, are currently aged 18 and over. However, the recent large-scale emigration of Venezuelans, particularly those in the young and productive age group, could potentially decrease the current population of individuals aged 18 and over in Venezuela. Additionally, the ongoing humanitarian crisis in Venezuela has resulted in a decline in life expectancy, which could further impact the proportion of individuals aged 18 and over. A conservative estimate suggests that there are currently between 22 and 23 million Venezuelans aged 18 and over. Therefore, with a 95% confidence level and a 5% margin of error, the sample size for this study was determined to be at least 384 participants. This was calculated using the Cochran formula. The sampling method employed was non-probabilistic.

### Survey design and data collection

A comprehensive survey was developed to assess the KAP related to the COVID-19 vaccine among vaccinated and unvaccinated individuals in Venezuela. The survey comprised 63 questions divided into five sections: sociodemographic characteristics, COVID-19 vaccination status, knowledge about the COVID-19 vaccine, attitudes towards the COVID-19 vaccine, and practices related to the COVID-19 vaccine (Supplementary Data 1). The sociodemographic characteristics section evaluated factors such as gender, age, marital status, education level, occupation, medical history, religion, residence, and income. The COVID-19 vaccination status section gathered information on the medium through which participants received information about the COVID-19 vaccine, their vaccination schedule, and their reasons for choosing to get vaccinated or not. Participants were then categorized into two groups based on their responses to the vaccination status section: those who had been vaccinated against COVID-19 and those who had not.

The knowledge section contained 12 items addressing various aspects of the COVID-19 vaccine such as its benefits, risks, efficacy, adverse effects, boosters, and specific conditions of administration. Each correct answer was awarded one point while incorrect answers received zero points, resulting in a total score ranging from 0 to 12 points. The attitudes section included 10 items concerning safety, benefits, confidence in the vaccine, efficacy, boosters, and available vaccines in Venezuela against COVID-19. Responses were recorded on a five-point Likert-type scale (1 = strongly disagree; 5 = strongly agree), with a total score range of 10 to 50 points. Lastly, the practices related to the COVID-19 vaccine section consisted of five items recorded on a five-point Likert scale (1 = never; 5 = always), with a total score range of 5 to 25 points.

### Survey validation and pilot test

The survey was designed and conducted by a team of three medical doctors and a psychologist, drawing upon relevant literature to ensure the rigor and readability of the questions. The survey underwent a validation process by a panel of medical professionals, including internists, infectologists, and epidemiologists, who evaluated its simplicity and relevance. Following expert qualitative validation, a pilot test was conducted with 120 participants, predominantly women (*n* = 65; 54.2%), with a mean age of 34 (SD —standard deviation— 15) years. This pilot test aimed to assess the clarity of the questions and their discriminative power. The “knowledge” dimension of the survey yielded dichotomous results (correct: 1; incorrect: 0), which were analyzed using the Two-Parameter Item Response Theory. To mitigate the impact of random responses, an “I don’t know” option was included in the “knowledge” dimension and subsequently analyzed as “incorrect”. Acceptable difficulty values ranged from −3 to +3, and discrimination values greater than 0.25 were considered acceptable. Under face validity, the items comprising the “knowledge” dimension demonstrated an appropriate level of discrimination (> 0.4) between those who know and those who do not, with varying difficulty levels (ranging from 0.1 to 0.8) (Supplementary Data 2).

The “attitudes” and “practices” dimensions were subjected to exploratory factor analysis using principal component analyses. The Kaiser-Meyer-Olkin test and Bartlett’s test of sphericity confirmed the suitability of the data for factor analysis, with a factor loading of ≥ 0.45 considered significant. In the “attitudes” dimension, reliability analysis revealed a Cronbach’s alpha of 0.916. Principal component factor analysis (Kaiser-Meyer-Olkin test = 0.923) resulted in two components. However, item 3 “SARS-CoV-2 exists (virus causing COVID-19)” was isolated. As this item does not compromise the overall reliability of the scale, it was retained. In the “practices” dimension, reliability analysis yielded a Cronbach’s alpha of 0.804. Principal component factor analysis (Kaiser-Meyer-Olkin test = 0.797) resulted in a single component. Item 5 “I get vaccinated annually against influenza/flu” should be approached with caution due to its low correlation with the scale. However, its inclusion does not significantly impact the overall reliability of the scale, so it was retained for analysis.

### Statistical analysis

Participant data were summarized using descriptive statistics, including mean, standard deviation (SD), median, interquartile range (IQR), frequency, and percentage (%). The distribution of numeric variables was assessed using the Kolmogorov–Smirnov test. For variables with a non-normal distribution, the Mann–Whitney U test was employed, while Student’s *t*-test was used for those with a normal distribution. Categorical variables were analyzed using Pearson’s chi-squared and Fisher’s exact tests. A *p*-value of less than 0.05 was considered statistically significant. In instances where *post-hoc* analysis was required, the Bonferroni correction was applied to adjust the *p*-value. To identify factors associated with high knowledge, positive attitudes, and appropriate practices among participants, a multinomial logistic regression (main effects) was used. The best fitting model was selected based on its goodness of fit, Nagelkerke’s pseudo R^2^, and Hosmer–Lemeshow test results. All statistical analyses were performed using the Statistical Package for the Social Sciences version 26 (IBM Corp., Armonk, NY, USA) and plotted with Microsoft® Excel® version 2019 (Microsoft, Redmond, WA, USA) and R version 4.2.2.

## Results

**Fig. 1:**
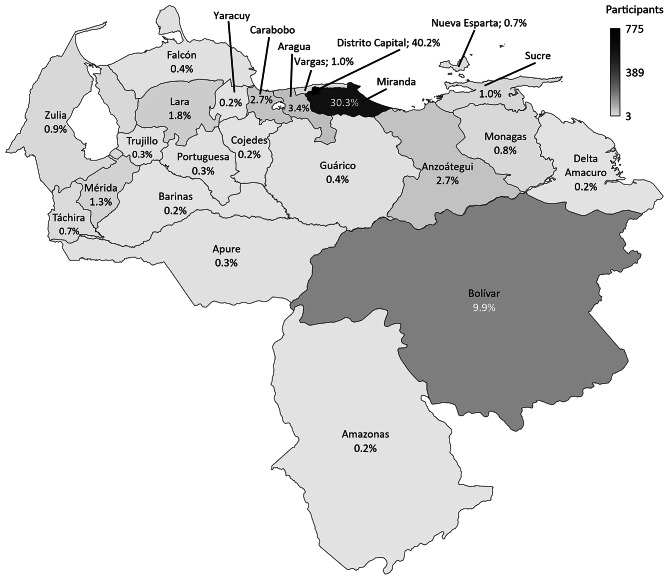
Participants from all 24 states of Venezuela included in the analysis. The number of participants surveyed is represented in grayscale. The percentage of participants surveyed is represented within each state

The analysis included a total of 1,930 participants residing across all 24 states of Venezuela (Fig. 1). The majority (93.4%, *n* = 1,802) reported being vaccinated against COVID-19, with the primary reasons for vaccination being “to protect myself against COVID-19” (81.5%) and “to protect my family, friends and/or neighbors against COVID-19” (72.4%). A total of 6.6% (*n* = 128) of the participants reported not being vaccinated against COVID-19, citing concerns about the safety of the COVID-19 vaccine for their health (58.6%) and lack of trust in the efficacy of the COVID-19 vaccines available in Venezuela (50.8%) as the main reasons for their decision (Supplementary Data 3).

The mean number of COVID-19 vaccine doses administered was 2.9 (SD 0.9), with most participants reporting having received two (33.8%) or three (39.7%) doses. A smaller percentage reported having received four (20.6%), five (3%), or a single dose (2.9%) of the vaccine. According to the National Immunization Commission led by the Venezuelan Society of Infectious Diseases and the *Ministerio del Poder Popular para la Salud* of Venezuela [7], only 37.7% of the participants had completed the COVID-19 vaccination schedule, considering their age and presence of comorbidities. Interestingly, nearly one-third of those vaccinated (30.1%, *n* = 542/1,802) admitted that they had to travel to another state to receive at least one dose of the COVID-19 vaccine.

The mean age of the participants was 40 (SD 17) years, with a majority being female (67.3%, *n* = 1,298), possessing a university education (70.6%, *n* = 1,362), and being employed (24.7%; *n* = 477). Additional sociodemographic characteristics of the participants are detailed in Table 1. A higher proportion of males were found among the unvaccinated participants compared to the vaccinated ones (40.6% vs. 31.9%, *p* = 0.037). Furthermore, having an associate degree and being self-employed were significantly associated with being unvaccinated (*p* < 0.001 for both), while being a healthcare worker was significantly associated with being vaccinated (*p* = 0.007) (Table 1).

**Table 1:** Sociodemographic characteristics and information sources among participants vaccinated and unvaccinated against COVID-19

Characteristics	Total (*n* = 1,930; 100%)	Vaccinated (*n* = 1,802; 93.4%)	Unvaccinated (*n* = 128; 6.6%)	*P*-value
Age, mean (SD), years	40 (17)	40 (17)	42 (16)	0.118^*^
Gender, *n* (%)				0.037^†^
Female	1,298 (67.3)	1,223 (67.9)	75 (58.6)	
Male	627 (32.5)	575 (31.9)	52 (40.6)	
Non-binary	5 (0.3)	4 (0.2)	1 (0.8)	
Marital status, *n* (%)				0.074^†^
Single	1,013 (52.5)	957 (53.1)	56 (43.8)	
Married	519 (26.9)	482 (26.7)	37 (28.9)	
Cohabiting (common-law)	210 (10.9)	189 (10.5)	21 (16.4)	
Divorced	129 (6.7)	117 (6.5)	12 (9.4)	
Widowed	59 (3.1)	57 (3.2)	2 (1.6)	
Education level, *n* (%)				0.005^‡§^
Primary school	2 (0.1)	2 (0.1)	0 (0)	
High school	388 (20.1)	363 (20.1)	25 (19.5)	
Associate degree	178 (9.2)	154 (8.5)	24 (18.8)	
University	1,362 (70.6)	1,283 (71.2)	79 (61.7)	
Occupation, *n* (%)				< 0.001^†||^
Employee	477 (24.7)	444 (24.6)	33 (25.8)	
Healthcare worker	470 (24.4)	454 (25.2)	16 (12.5)	
Self-employed	402 (20.8)	357 (19.8)	45 (35.2)	
Student	394 (20.4)	374 (20.8)	20 (15.6)	
Retired	142 (7.4)	131 (7.3)	11 (8.6)	
Unemployed	45 (2.3)	42 (2.3)	3 (2.3)	
Monthly income, *n* (%)				0.524^†^
≤$100	563 (29.2)	521 (28.9)	42 (32.8)	
$101–200	427 (22.1)	396 (22)	31 (24.2)	
$201–300	253 (13.1)	236 (13.1)	17 (13.3)	
>$300	687 (35.6)	649 (36)	38 (29.7)	
Information sources about the COVID-19 vaccine, yes (%)				
Internet (Google information search)	1,127 (58.4)	1042 (57.8)	85 (66.4)	0.057^†^
Advice from healthcare workers	1,037 (53.7)	989 (54.9)	48 (37.5)	< 0.001^†^
Journals and/or scientific articles	819 (42.4)	770 (42.7)	49 (38.3)	0.325^†^
Instagram	611 (31.7)	569 (31.6)	42 (32.8)	0.771^†^
Television	527 (27.3)	490 (27.2)	37 (28.9)	0.674^†^
Twitter	415 (21.5)	386 (21.4)	29 (22.7)	0.742^†^
Educational lectures	393 (20.4)	369 (20.5)	24 (18.8)	0.639^†^
Advice from family, friends and/or neighbors	391 (20.3)	355 (19.7)	36 (28.1)	0.022^†^
YouTube	274 (14.2)	242 (13.4)	32 (25)	< 0.001^†^
Radio	205 (10.6)	185 (10.3)	20 (15.6)	0.057^†^
Newspapers	149 (7.7)	130 (7.2)	19 (14.8)	0.002^†^
Facebook	144 (7.5)	130 (7.2)	14 (10.9)	0.121^†^
TikTok	81 (4.2)	72 (4)	9 (7)	0.098^†^
Other	36 (1.9)	30 (1.7)	6 (4.7)	0.015^†^

^*^Student’s *t*-test for independent samples; ^†^Pearson’s chi-square test; ^‡^Fisher’s exact test; ^§^Significant only for Associate degree (*p* < 0.001) for a value of α ≤ 0.01 by Bonferroni correction; ^||^Significant only for healthcare worker (*p* = 0.007) and for Independent (*p* < 0.001) for a value of α ≤ 0.008 by Bonferroni correction. Participants who responded “non-binary” were excluded from the gender analysis only on this occasion

### Information sources on COVID-19 vaccine

The most frequently used source of information about the COVID-19 vaccine among participants was Internet search (58.4%, *n* = 1,127), followed by advice from healthcare workers (53.7%, *n* = 1,037), journals and/or scientific articles (42.4%, *n* = 819), and Instagram (31.7%, *n* = 611). Additional sources of information about the COVID-19 vaccine are detailed in Table 1. A higher proportion of vaccinated participants reported receiving advice from healthcare workers as a source of information about the COVID-19 vaccine compared to unvaccinated participants (54.9% vs. 37.5%, *p* < 0.001). Conversely, a higher proportion of unvaccinated participants reported receiving advice from family, friends and/or neighbors, using newspapers, and YouTube as sources of information about the COVID-19 vaccine compared to vaccinated participants (28.1% vs. 19.7%, *p* = 0.022; 25% vs. 13.4%, *p* < 0.001; 14.8% vs. 7.2%, *p* = 0.002; respectively) (Table 1).

### Knowledge about the COVID-19 vaccine

Knowledge about the COVID-19 vaccine was categorized into three levels: low (≤ 7 points), moderate (8–9 points), and high (≥ 10 points). The mean knowledge score was 7.5 (SD 2.5) points, with a higher score observed in the vaccinated group compared to the unvaccinated group (7.79 vs. 3.94 points, *p* < 0.001). The majority of participants (43%, *n* = 830) demonstrated low knowledge, predominantly among the unvaccinated compared to the vaccinated group (94.5% vs. 39.3%, *p* < 0.001). The proportion of correct answers was significantly higher in the vaccinated group compared to the unvaccinated group for almost all knowledge questions. The questions with the lowest proportion of correct responses were those related to the possibility of contracting COVID-19 from vaccination (35%), the efficacy of available COVID-19 vaccines against newer variants of the virus (34.8%), and the recommended age to start vaccination (25.5%) (Table 2).

**Table 2:** Knowledge about the COVID-19 vaccine among participants vaccinated and unvaccinated against COVID-19

Knowledge	Total (*n* = 1,930; 100%)	Vaccinated (*n* = 1,802; 93.4%)	Unvaccinated (*n* = 128; 6.6%)	*P*-value
Knowledge, mean (SD), points	7.5 (2.5)	7.79 (2.33)	3.94 (1.95)	< 0.001^*^
Knowledge, *n* (%)				
Low (≤ 7 points)	830 (43.0)	709 (39.3)	121 (94.5)	< 0.001^†^
Moderate (8–9 points)	65 (33.7)	643 (35.7)	7 (5.5)	< 0.001^†^
High (≥ 10 points)	450 (23.3)	450 (25)	0 (0)	< 0.001^†^
COVID-19 vaccine decreases the risk of developing severe COVID-19 and dying, correct (%)	1,596 (82.7)	1,570 (87.1)	26 (20.3)	< 0.001^†^
COVID-19 vaccine helps protect the community against the virus, correct (%)	1,565 (81.1)	1,542 (85.6)	23 (18)	< 0.001^†^
It is possible to become sick with COVID-19 because of vaccination, correct (%)	675 (35)	658 (36.5)	17 (13.3)	< 0.001^†^
The COVID-19 vaccine may cause minor side effects, such as fatigue, fever, and malaise, correct (%)	1,745 (90.4)	1,642 (91.1)	103 (80.5)	< 0.001^†^
Some COVID-19 vaccines are more effective than others, correct (%)	1,024 (53.1)	995 (55.2)	29 (22.7)	< 0.001^†^
Available COVID-19 vaccines are less effective against newer variants of the virus (e.g., Omicron), correct (%)	672 (34.8)	632 (35.1)	40 (31.3)	0.38^†^
Vaccination against COVID-19 has more risks than benefits, correct (%)	1,350 (69.9)	1,339 (74.3)	11 (8.6)	< 0.001^†^
Booster doses of COVID-19 vaccine increase protection against the virus, correct (%)	1,424 (73.8)	1,407 (78.1)	17 (13.3)	< 0.001^†^
It is recommended that people with risk factors for developing severe COVID-19, such as hypertension and diabetes, be vaccinated against COVID-19, correct (%)	1,550 (80.3)	1,504 (83.5)	46 (35.9)	< 0.001^†^
Natural immunity (catching the virus) may be boosted with the COVID-19 vaccine, correct (%)	1,157 (59.9)	1,136 (63)	21 (16.4)	< 0.001^†^
Starting at six months of age, all persons may receive the COVID-19 vaccine, such as Pfizer or Moderna, correct (%)	492 (25.5)	477 (26.5)	15 (11.7)	< 0.001^†^
Pregnant women may be vaccinated against COVID-19, correct (%)	1,071 (55.5)	1,040 (57.7)	31 (24.2)	< 0.001^†^

^*^Student’s *t*-test for independent samples; ^†^Pearson’s chi-square test

### Attitudes towards the COVID-19 vaccine

**Fig. 2:**
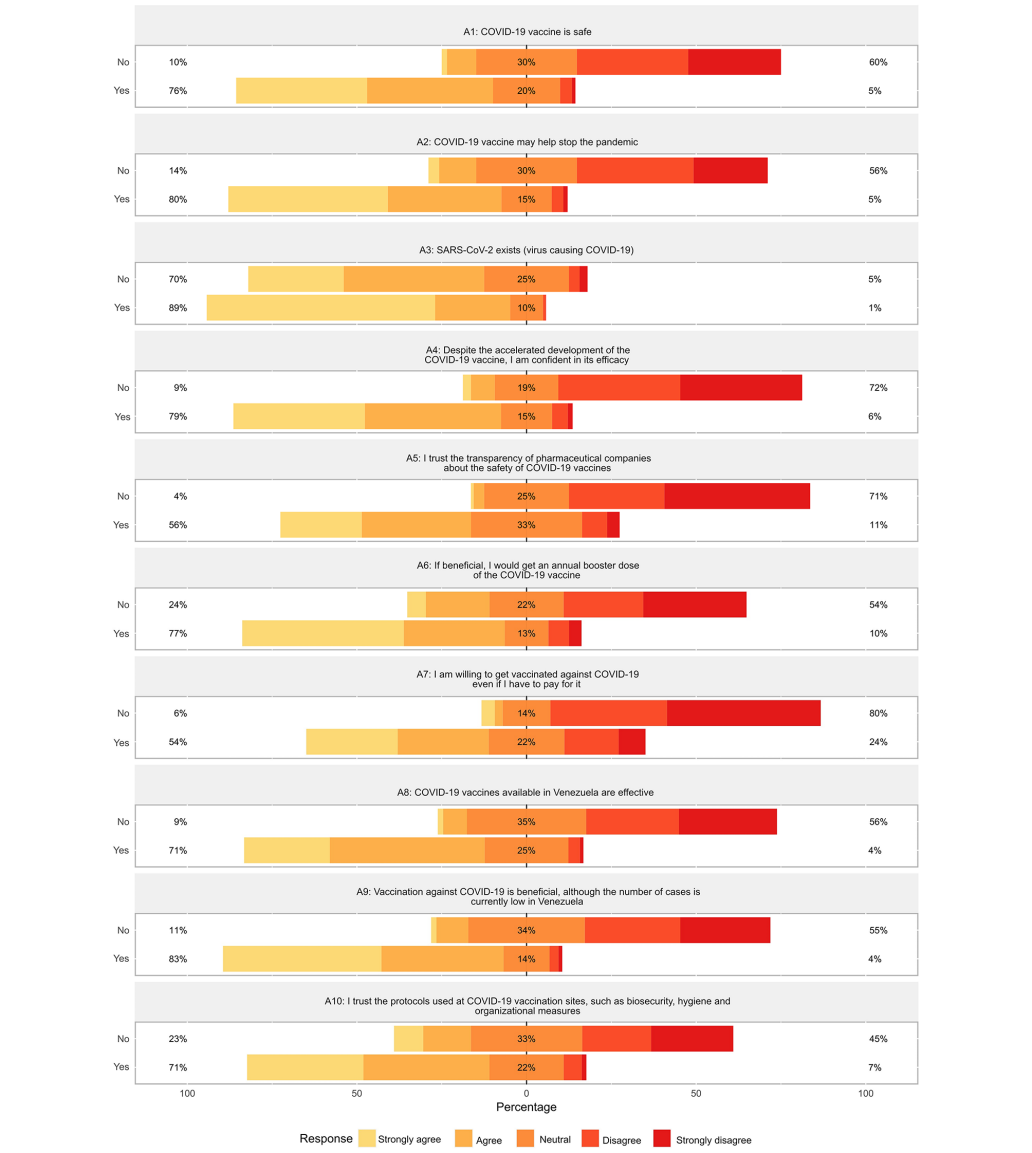
Attitudes towards the COVID-19 vaccine among vaccinated and unvaccinated participants. The bars indicate the proportion of participants based on their COVID-19 vaccination status. Positive attitudes are represented towards the left, while negative attitudes are represented towards the right

Attitudes towards the COVID-19 vaccine were categorized into three levels: negative (≤ 29 points), indifferent (30–39 points), and positive (≥ 40 points). The mean attitude score was 39 (SD 8) points, with a higher score observed in the vaccinated group compared to the unvaccinated group (40 vs. 24 points, *p* < 0.001). Positive attitudes were prevalent among the majority of participants (55.1%, *n* = 1,063), particularly among the vaccinated compared to the unvaccinated group (58.9% vs. 0.8%, *p* < 0.001). Most participants expressed belief in the safety of the COVID-19 vaccine (71.3% strongly agreed or agreed, *n* = 1,375), its potential to help stop the pandemic (75.9%, *n* = 1,466), and confidence in its efficacy despite its accelerated development (74.3%, *n* = 1,433); these positive trends were significantly higher among vaccinated participants (*p* < 0.001). Furthermore, confidence in the protocols used at COVID-19 vaccination sites, including biosecurity, hygiene, and organizational measures, was prevalent (68.1%, *n* = 1,316) and significantly higher among vaccinated participants (*p* < 0.001). However, at least one in four participants expressed unwillingness to get vaccinated against COVID-19 if they had to pay for it (27.7% strongly disagreed or disagreed, *n* = 533), with this negative trend being significantly higher among unvaccinated participants (*p* < 0.001). Additional attitudes may be seen in Fig. 2 and Supplementary Data 4.

### Practices related to the COVID-19 vaccine

**Fig. 3:**
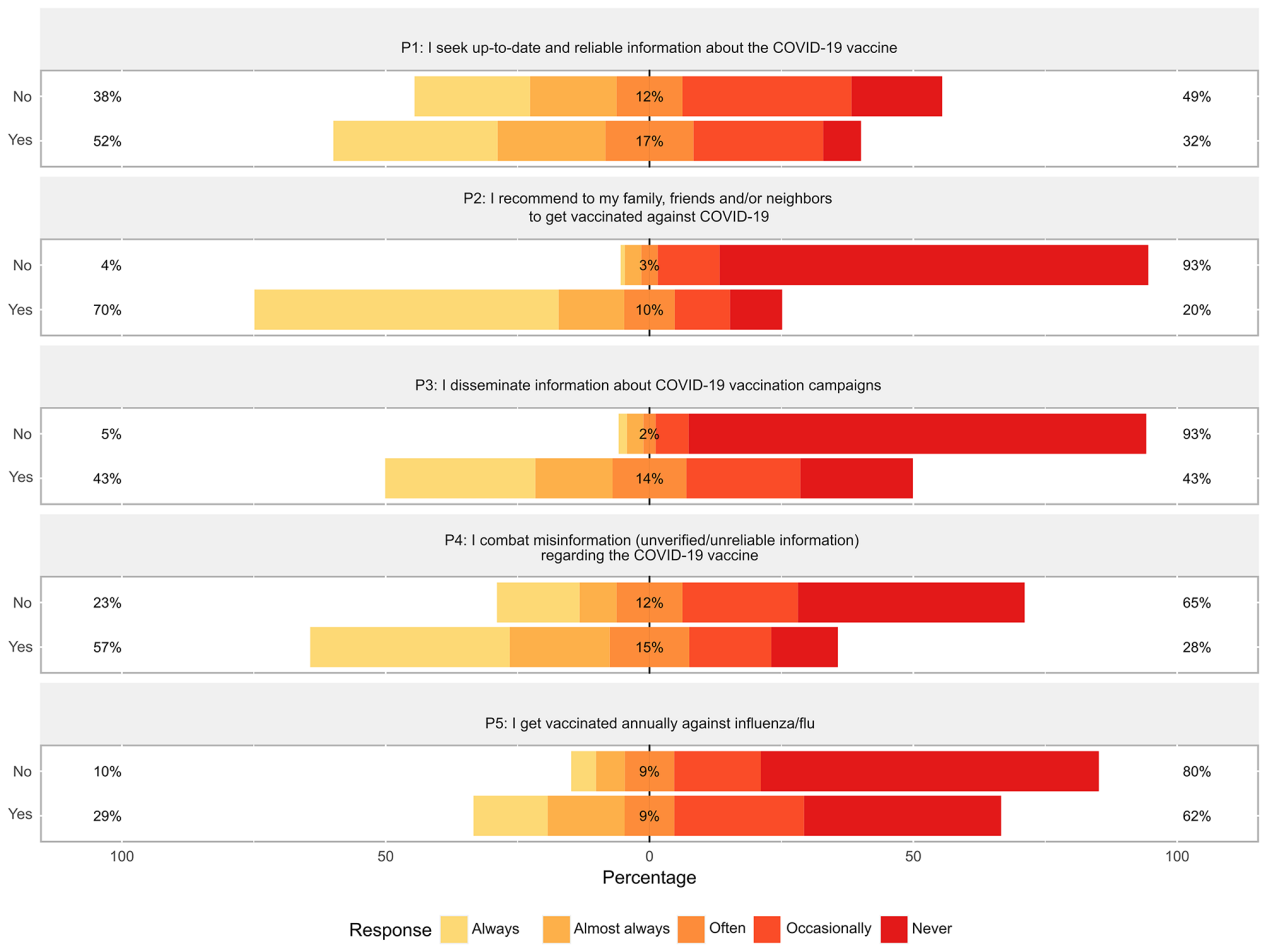
Practices related to the COVID-19 vaccine among vaccinated and unvaccinated participants. The bars indicate the proportion of participants based on their COVID-19 vaccination status. Appropriate practices are represented towards the left, while inappropriate practices are represented towards the right

Practices related to the COVID-19 vaccine were categorized into three levels: inappropriate (≤ 14 points), indifferent (15–19 points), and appropriate (≥ 20 points). The mean practice score was 16 (SD 6) points, with a higher score observed in the vaccinated compared to the unvaccinated group (16 vs. 10 points, *p* < 0.001). Inappropriate practices were prevalent among the majority of participants (41.4%, *n* = 799), particularly among the unvaccinated compared to the vaccinated group (91.4% vs. 37.8%, *p* < 0.001). While most participants always or almost always sought up-to-date and reliable information about the COVID-19 vaccine (50.6%, *n* = 977), recommended vaccination to their family, friends, and/or neighbors (65.6%, *n* = 1,267), and combated misinformation regarding the vaccine (54.4%, *n* = 1,051), a significant proportion did not disseminate information about vaccination campaigns (25.8%, *n* = 497) or did so only occasionally (20.6%, *n* = 397). This trend was significantly more prevalent among unvaccinated participants (*p* < 0.001). Furthermore, only a minority of participants reported always or almost always getting vaccinated annually against influenza/flu (27.2%, *n* = 497). Additional practices may be seen in Fig. 3 and Supplementary Data 5.

### Factors associated with KAP outcomes

In relation to COVID-19 vaccination status, our findings indicate that a 1-point increase in the “knowledge” dimension increased the odds of being vaccinated by 84.6% (β = 0.613, *p* < 0.001). Similarly, a 1-point increase in the “attitudes” dimension increased the odds of being vaccinated by 25.6% (β = 0.228, *p* < 0.001), while a 1-point increase in the “practices” dimension increased the odds of being vaccinated by 33% (β = 0.286, *p* < 0.001).

Regarding the knowledge model about the COVID-19 vaccine (goodness of fit = 0.677, Nagelkerke’s pseudo R^2^ = 0.31, *p* < 0.001), it was found that, compared to high knowledge, being single (adjusted OR = 0.305, 95% CI —confidence interval—= 0.108–0.862, *p* = 0.025) or married (adjusted OR = 0.274, 95% CI = 0.101–0.743, *p* = 0.011), being student (adjusted OR = 0.318, 95% CI = 0.181–0.561, *p* < 0.001) or healthcare worker (adjusted OR = 0.084, 95% CI = 0.054–0.132, *p* < 0.001), practicing atheism (adjusted OR = 0.278, 95% CI = 0.083–0.927, *p* = 0.037), and having a monthly income greater than $300 (adjusted OR = 0.495, 95% CI = 0.343–0.713, *p* < 0.001) decreased the probability of having low knowledge, while having an associate degree (adjusted OR = 3.161, 95% CI = 1.71–5.845, *p* < 0.001) and residing in Carabobo (adjusted OR = 8.686, 95% CI = 1.699–44.406, *p* = 0.009), Merida (adjusted OR = 8.43, 95% CI = 1.446–49.133, *p* = 0.018), or Sucre (adjusted OR = 8.337, 95% CI = 1.073–64.76, *p* = 0.043) states increased the probability of having low knowledge. In the attitudes model (goodness of fit = 1, Nagelkerke’s pseudo R^2^ = 0.339, *p* < 0.001), it was found that, compared to positive attitudes, being a healthcare worker (adjusted OR = 0.324, 95% CI = 0.179–0.586, *p* < 0.001), not having chronic kidney disease (adjusted OR = 0.041, 95% CI = 0.005–0.309, *p* = 0.002), asthma (adjusted OR = 0.29, 95% CI = 0.135–0.624, *p* = 0.002), or any comorbidity (adjusted OR = 0.408, 95% CI = 0.199–0.836, *p* = 0.014), and having a monthly income greater than $300 (adjusted OR = 0.533, 95% CI = 0.323–0.881, *p* = 0.014) decreased the probability of having negative attitudes, while being young (adjusted OR = 1.019, 95% CI = 1.001–1.038, *p* = 0.043), not being vaccinated (adjusted OR = 863.127, 95% CI = 116.765–6380.247, *p* < 0.001), and residing in Sucre state (adjusted OR = 12.003, 95% CI = 1.169–123.199, *p* = 0.036) increased the probability of having negative attitudes. Finally, in the practices model (goodness of fit = 0.21, Nagelkerke’s pseudo R^2^ = 0.302, *p* < 0.001), it was found that, compared to appropriate practices, advance age (adjusted OR = 0.97, 95% CI = 0.957–0.983, *p* < 0.001), being a healthcare worker (adjusted OR = 0.094, 95% CI = 0.062–0.143, *p* < 0.001), and having a monthly income greater than $300 (adjusted OR = 0.653, 95% CI = 0.461–0.924, *p* = 0.016) decrease the odds of having inappropriate practices, while residing in La Guaira state (adjusted OR = 20.776, 95% CI = 1.251–345.101, *p* = 0.034) increases the odds of having inappropriate practices (Table 3).

**Table 3:** Factors associated with KAP outcomes among vaccinated and unvaccinated Venezuelan participants against COVID-19

Factors	β	*P*-value	Adjusted OR (95% CI)
*Low knowledge*			
Marital status: Married	-1.294	0.011	0.274 (0.101–0.743)
Marital status: Single	-1.188	0.025	0.305 (0.108–0.862)
Education level: Associate degree	1.151	< 0.001	3.161 (1.71–5.845)
Occupation: Student	-1.145	< 0.001	0.318 (0.181–0.561)
Occupation: Healthcare worker	-2.472	< 0.001	0.084 (0.054–0.132)
Religion: Atheist	-1.282	0.037	0.278 (0.083–0.927)
State: Carabobo	2.162	0.009	8.686 (1.699–44.406)
State: Merida	2.132	0.018	8.43 (1.446–49.133)
State: Sucre	2.121	0.043	8.337 (1.073–64.76)
Monthly income: >$300	-0.704	< 0.001	0.495 (0.343–0.713)
*Moderate knowledge*			
Marital status: Married	-1.042	0.04	0.353 (0.13–0.953)
Education level: Associate degree	0.765	0.017	2.149 (1.146–4.029)
Occupation: Student	-0.592	0.036	0.553 (0.318–0.961)
Occupation: Healthcare worker	-1.304	< 0.001	0.271 (0.181–0.408)
*Negative attitudes*			
Age	0.019	0.043	1.019 (1.001–1.038)
Not vaccinated	6.761	< 0.001	863.127 (116.765–6380.247)
Occupation: Healthcare worker	-1.127	< 0.001	0.324 (0.179–0.586)
Pathological history: no chronic kidney disease	-3.194	0.002	0.041 (0.005–0.309)
Pathological history: no asthma	-1.238	0.002	0.29 (0.135–0.624)
Pathological history: None	-0.896	0.014	0.408 (0.199–0.836)
State: Sucre	2.485	0.036	12.003 (1.169–123.199)
Monthly income: >$300	-0.628	0.014	0.533 (0.323–0.881)
*Indifferent attitudes*			
Age	-0.013	0.032	0.987 (0.976–0.999)
Not vaccinated	3.833	< 0.001	46.221 (6.187–345.304)
Education level: High school	-0.36	0.038	0.698 (0.497–0.98)
Education level: Associate degree	-0.573	0.009	0.564 (0.367–0.867)
Occupation: Healthcare worker	-0.891	< 0.001	0.41 (0.291–0.579)
Pathological history: no hypertension	-0.513	0.019	0.599 (0.39–0.919)
Pathological history: no chronic kidney disease	-1.833	0.045	0.16 (0.027–0.963)
Pathological history: no asthma	-0.584	0.018	0.557 (0.343–0.905)
State: Lara	1.674	0.028	5.336 (1.197–23.789)
State: Merida	1.873	0.022	6.508 (1.31–32.336)
State: Sucre	2.317	0.008	10.145 (1.838–56.01)
Monthly income: >$300	-0.634	< 0.001	0.53 (0.394–0.714)
*Inappropriate practices*			
Age	-0.031	< 0.001	0.97 (0.957–0.983)
Occupation: Healthcare worker	-2.362	< 0.001	0.094 (0.062–0.143)
State: La Guaira	3.034	0.034	20.776 (1.251–345.101)
Monthly income: >$300	-0.426	0.016	0.653 (0.461–0.924)
*Indifferent practices*			
Occupation: Retired	0.602	0.039	1.826 (1.032–3.229)
Occupation: Employee	0.599	0.004	1.82 (1.207–2.743)
Occupation: Healthcare worker	-0.989	< 0.001	0.372 (0.249–0.555)
Pathological history: no obesity	0.576	0.027	1.779 (1.066–2.968)

The reference categories are High Knowledge for Knowledge, Positive Attitudes for Attitudes, and Appropriate Practices for Practices

### KAP associations

When crossing the KAP about the COVID-19 vaccine with each other, in terms of the knowledge model (goodness of fit = 0.481, Nagelkerke’s pseudo R^2^ = 0.328, *p* < 0.001), it was found that, compared to high knowledge, having negative (adjusted OR = 41.475, 95% CI = 12.804–134.345, *p* < 0.001) or indifferent (adjusted OR = 4.783, 95% CI = 3.438–6.645, *p* < 0.001) attitudes, and having inappropriate (adjusted OR = 8.72, 95% CI = 5.95–12.781, *p* < 0.001) or indifferent (adjusted OR = 3.168, 95% CI = 2.251–4.458, *p* < 0.001) practices increased the odds of having low knowledge. In the attitudes model (goodness of fit = 0.418, Nagelkerke’s pseudo R^2^ = 0.399, *p* < 0.001), it was found that, compared to positive attitudes, having low knowledge (adjusted OR = 41.475, 95% CI = 12.804–134.345, *p* < 0.001), and having inappropriate (adjusted OR = 72.142, 95% CI = 22.406–232.273, *p* < 0.001) or indifferent (adjusted OR = 3.779, 95% CI = 1.049–13.608, *p* = 0.042) practices increased the odds of having negative attitudes. Finally, in the practices model (goodness of fit = 0.418, Nagelkerke’s pseudo R^2^ = 0.365, *p* < 0.001), it was found that, compared with appropriate practices, having low (adjusted OR = 8.72, 95% CI = 5.95–12.781, *p* < 0.001) or moderate (adjusted OR = 2.914, 95% CI = 2.044–4.155, *p* < 0.001) knowledge, and having negative (adjusted OR = 72.142, 95% CI = 22.406–232.273, *p* < 0.001) or indifferent (adjusted OR = 8.191, 95% CI = 6.074–11.046, *p* < 0.001) attitudes increased the odds of having inappropriate practices (Table 4).

**Table 4:** KAP associations among vaccinated and unvaccinated Venezuelan participants against COVID-19

Factors	β	*P*-value	Adjusted OR (95% CI)
*Low knowledge*			
Negative attitudes	3.725	< 0.001	41.475 (12.804–134.345)
Indifferent attitudes	1.565	< 0.001	4.783 (3.438–6.654)
Inappropriate practices	2.166	< 0.001	8.72 (5.95–12.781)
Indifferent practices	1.153	< 0.001	3.168 (2.251–4.458)
*Moderate knowledge*			
Indifferent attitudes	0.679	< 0.001	1.971 (1.426–2.724)
Inappropriate practices	1.069	< 0.001	2.914 (2.044–4.155)
Indifferent practices	0.648	< 0.001	1.912 (1.444–2.531)
*Negative attitudes*			
Inappropriate practices	4.279	< 0.001	72.142 (22.406–232.273)
Indifferent practices	1.329	0.042	3.779 (1.049–13.608)
Low knowledge	3.725	< 0.001	41.475 (12.804–134.345)
*Indifferent attitudes*			
Inappropriate practices	2.103	< 0.001	8.191 (6.074–11.046)
Indifferent practices	0.706	< 0.001	2.025 (1.489–2.754)
Low knowledge	1.565	< 0.001	4.783 (3.438–6.654)
Moderate knowledge	0.679	< 0.001	1.971 (1.426–2.724)
*Inappropriate practices*			
Low knowledge	2.166	< 0.001	8.72 (5.95–12.781)
Moderate knowledge	1.069	< 0.001	2.914 (2.044–4.155)
Negative attitudes	4.279	< 0.001	72.142 (22.406–232.273)
Indifferent attitudes	2.103	< 0.001	8.191 (6.074–11.046)
*Indifferent practices*			
Low knowledge	1.153	< 0.001	3.168 (2.251–4.458)
Moderate knowledge	0.648	< 0.001	1.912 (1.444–2.531)
Negative attitudes	1.329	0.042	3.779 (1.049–13.608)
Indifferent attitudes	0.706	< 0.001	2.025 (1.489–2.754)

The reference categories are High Knowledge for Knowledge, Positive Attitudes for Attitudes, and Appropriate Practices for Practices

## Discussion

Prior research has attempted to establish a correlation between demographic factors and hesitancy towards COVID-19 vaccination. The existing literature predominantly portrays Venezuela in a pre-vaccine era, as the studies were conducted during the first quarter of 2021. During this period, COVID-19 vaccines were in the nascent stages of distribution to the general populace. Consequently, the focus of these studies was primarily on the intent to get vaccinated rather than the act of vaccination itself. A significant limitation of these studies was the prevailing uncertainty regarding vaccine accessibility [1]. Therefore, understanding the perception of vaccines after their widespread distribution and improved accessibility could offer valuable insights into the KAP of the Venezuelan population towards COVID-19 vaccination.

### Sociodemographics and COVID-19 vaccination status

This study revealed sociodemographic differences between the COVID-19 vaccinated and unvaccinated participant groups. The proportion of vaccinated participants in our study was higher (93%) compared to the WHO’s data for Venezuela (78%). This discrepancy may be attributed to a larger representation of healthcare workers in our sample, a demographic known to exhibit a higher propensity for vaccination [8]. Our findings also identified that the vaccinated group predominantly consisted of females, college-educated individuals, with high income, while the unvaccinated group had a higher proportion of males, individuals with low education, and low income. These findings coincide with an international study that reported socioeconomic inequalities in vaccination coverage across 19 countries [9]. Another study also demonstrated lower vaccination coverage in groups with lower income and education [10]. Interestingly, the vaccinated group reported a higher frequency of medical history. This could be attributed to the influence of health status on the perception of risk and decision to get vaccinated, as demonstrated in a study that found people who perceived a high or very high risk of infection had a greater intention to be vaccinated [11]. Despite these demographic findings, other studies have reported increased vaccination hesitancy among females, urban populations, and individuals with higher education. Therefore, health promotion efforts should be directed towards the general population to address these concerns [12, 13].

Despite reports from the Pan American Health Organization (PAHO) indicating that only 2.3% of the Venezuelan population had received any booster dose by June 2022 [14], almost 40% of participants in our sample had received at least one booster dose at the time of the study. This discrepancy could be due to differences between weekly reporting to PAHO and actual vaccination speed [15]. However, a decrease in the percentage of vaccinated persons was observed after the first booster dose (third dose), and less than a quarter of participants completed the vaccination schedule recommended by the *Ministerio del Poder Popular para la Salud* of Venezuela, which consists of four doses by July 2022 [7]. Factors that could explain refusal to receive booster doses include doubts about their efficacy and safety [16], low educational level, food insecurity or having had COVID-19 previously [17]. Additionally, a phenomenon known as pandemic fatigue [18], which refers to mental and physical exhaustion caused by COVID-19-related restrictions, was detected. This could lead some people to take fewer precautions to prevent the spread of the virus.

### Knowledge about the COVID-19 vaccine and its relationship to vaccine uptake

Our study found that the majority of participants had a low to moderate level of knowledge about the COVID-19 vaccine, a finding that contrasts with studies conducted in countries such as Greece [19], Singapore [8], Ethiopia [20], and Malaysia [21], where higher levels of knowledge were reported. This discrepancy could be attributed to inefficient communication and access to vaccination campaigns in Venezuela, as the questions with the lowest scores pertained to COVID-19 variants and vaccination protocols for specific groups (children and pregnant women), topics that should be disseminated through official channels. However, Venezuela has not adhered to WHO recommendations on trust-based risk communication and transparency of vaccination data, being the only South American country without a strategic report on its vaccination plan to the Inter-American Commission on Human Rights [15]. Additionally, the accessibility of vaccination-related information, particularly in languages other than English, and the widespread presence of misinformation on social networks and online platforms, are additional factors contributing to vaccine hesitancy, as evidenced in other online survey studies [13].

According to a national study [15], only 38% of the country’s parishes had assigned vaccination centers (2.21 centers per 100,000 inhabitants), implying that at least 839,733 Venezuelans did not have vaccination centers in their locality, thus hindering their access to information and vaccination. Despite this scenario, the population demonstrated a basic level of knowledge about the degree of protection offered by the COVID-19 vaccine, which could be associated with a high vaccination rate. In the absence of effective official information channels, Venezuelans resorted to various sources. Among the vaccinated, advice from healthcare workers and internet searches were most commonly used, while among the unvaccinated, platforms such as YouTube, newspapers, and advice from family/friends predominated.

In this study, unvaccinated individuals were found to have a low level of knowledge, contrasting with reports from Singapore where higher awareness and acceptance of vaccination were observed [8, 22–25]. The level of knowledge is a determinant of willingness to vaccinate, as it influences the perceived risk versus benefit of vaccination. From a public health perspective, enhancing the population’s health literacy is deemed a strategic tool for disease prevention [26]. Therefore, it is crucial to assess the relationship between knowledge and vaccination status in populations such as Venezuela, which are susceptible to misinformation. Additionally, the efficacy and safety of the COVID-19 vaccine [27], its boosters [28, 29] and heterologous vaccination [29–31] have been demonstrated.

### Attitudes towards the COVID-19 vaccine and their relationship to vaccine acceptance

This study explored participants’ attitudes towards the COVID-19 vaccine and the factors influencing them. The majority (55.1%) showed a positive attitude towards vaccination, consistent with studies conducted in countries such as Lebanon (52.8%) [32], Oman (59.3%) [33], Ethiopia (47.1%) [20], and Bosnia and Herzegovina (49.6%) [34]. However, a significant level of neutrality was observed in relation to trust in the pharmaceutical company producing the vaccine and willingness to be vaccinated if they had to pay for it. This could suggest a lack of credibility in pharmaceutical companies and distrust of the speed at which vaccines were developed, aspects identified as important factors in vaccine refusal in other studies [35–37].

The study also found that the majority (80.3%) of participants agreed to be vaccinated, consistent with other studies conducted in low- and middle-income countries [24, 38–40]. A high prevalence of positive attitude towards vaccination (58.9%) was observed among the vaccinated, whereas only 0.8% of the unvaccinated exhibited a positive attitude. The vaccinated scored higher on all attitude items than the unvaccinated. Additionally, it was estimated that for each point increase in the attitude scale score, the probability of being vaccinated increases by 25.6%. These results are consistent with studies conducted in Singapore [8] and Bangladesh [41], supporting previous evidence that a positive attitude towards vaccination is directly related to the decision to be vaccinated [39]. Furthermore, it was evidenced that the relationship between knowledge and attitudes depend on the quality of information received [33]. Large-scale global studies suggest similar findings, emphasizing how factors such as vaccine availability, healthcare worker recommendation, and vaccine cost play pivotal roles in deciding whether to get vaccinated against COVID-19 [24, 25]. Science-based information about the manufacture, benefits, and potential adverse effects of the COVID-19 vaccine may modify internalized beliefs and increase willingness to vaccinate. These findings underscore the importance of promoting accurate and scientific information about the COVID-19 vaccine to improve attitudes and increase acceptance of vaccination in the population.

### Practices related to the COVID-19 vaccine and their relationship to vaccine uptake

In this study, inappropriate practices were observed more frequently in the unvaccinated compared to the vaccinated group. This finding contrasts with a study from Singapore, where both groups exhibited a similar frequency of such practices [8]. One potential explanation for this discrepancy could be the higher proportion of healthcare workers in the vaccinated group, which could provide greater protection against inappropriate practices [8]. However, it was also noted that a proportion of Venezuelan healthcare workers had not been vaccinated. Factors associated with willingness to be vaccinated in this population include young age, female sex, and employment in the public system [42]. Contrary to other studies [8], this study found that a high score in the “practices” dimension increased the probability of being vaccinated by 33%.

A significant difference was observed between the vaccinated and unvaccinated groups in terms of recommending the COVID-19 vaccine to their relatives, friends and/or neighbors and disseminating information about vaccination campaigns, as reported in a study in Bangladesh [41]. Older age was identified as a factor that reduces the odds of inappropriate practices. This aligns with results from studies conducted in Bangladesh, Colombia, India, Malaysia, Zimbabwe, and the USA [43], where the age group 65–74 years was found to be a positive predictor for the development of good practices. These findings underscore the importance of promoting good practices and considering factors such as age and occupation when designing strategies to improve compliance and increase willingness to vaccinate.

### Factors associated with KAP outcomes

The study revealed an inverse association between monthly income and low knowledge, negative attitudes, and inappropriate practices about vaccination, consistent with previous studies conducted in England [44, 45] and Switzerland [46, 47]. This variable is suggested to have an indirect effect as it determines the socioeconomic environment of the population and its opportunities. It is argued that individuals with higher economic status have access to more information and resources, especially medical care that provides guidance on vaccination. Similarly, belonging to the healthcare workers is also positively related to willingness to be vaccinated [8], likely due to these workers having a higher risk of exposure to the virus and thus greater motivation to be vaccinated. Additionally, they may be more informed about the safety and efficacy of the vaccine due to their experience in the health setting.

Interestingly, the absence of chronic diseases was associated with a positive attitude towards vaccines. While asthmatic patients were assumed to be at higher risk of severe complications from common viral infections, recent studies indicate that these patients do not have increased severity from SARS-CoV-2 and may even have a protective factor [48–50]. Renal disease is a risk factor associated with suboptimal responses to COVID-19 vaccination and increased mortality from COVID-19. Uncertainty about the efficacy of vaccination in this group of patients may explain why healthy patients were found to have better attitudes towards vaccination than patients with chronic kidney disease [51, 52].

Residing in regions other than the capital was found to be associated with low knowledge (Merida, Sucre, Carabobo), inappropriate attitudes (Sucre), and inappropriate practices (La Guaira) about vaccination. A national study [15] estimated this inequality in vaccine distribution, showing that the government allocated 43% more centers to the 30 richest municipalities in the country than to the 30 poorest. It also pointed out that Bolivar, Vargas, and Carabobo were the states with the lowest number of vaccination centers per inhabitant, which could explain the relevance of these states in anti-vaccination behavior. Furthermore, Sucre was declared one of the poorest eastern states of the country by ENCOVI in July 2021, with 83% of its population below the poverty line [6], highlighting the impact of economic variables on anti-vaccine attitudes in this region. This finding coincides with demographic studies where poverty is identified as a significant determinant of non-vaccination, emphasizing the need for targeted interventions in these areas [53, 54].

### KAP associations

This study investigates the interplay between KAP in relation to COVID-19 vaccination. The findings suggest that individuals with limited knowledge are more prone to harbor negative attitudes towards vaccination. This could be attributed to the fact that inadequate information about the vaccine may foster doubts regarding its safety or necessity. These results align with studies conducted in Oman [33] and Bosnia [55], where a significant correlation was observed between higher knowledge levels and positive attitudes towards vaccination. In line with other studies [55, 56], participants with higher knowledge scores were significantly more likely to participate in vaccination programs. Similarly, this study found that individuals with lower knowledge levels or negative attitudes were more likely to demonstrate negative or indifferent practices towards vaccination. This could stem from the development of prejudices and negative perceptions about vaccination, compounded by a lack of adequate information, which directly impacts the willingness to engage in vaccination-related activities. These findings underscore the importance of enhancing public knowledge about vaccination to foster positive attitudes and appropriate practices.

### Limitations

This study has several noteworthy limitations, primarily related to the employment of non-probabilistic sampling. Firstly, the sample was not representative of some of the 24 Venezuelan states included, due to the small number of participants. Moreover, 90% of the responses corresponded to Venezuelan participants vaccinated against COVID-19, indicating a potential undercoverage bias and increasing the likelihood of a non-representative sample of the unvaccinated group. According to the registry dated November 26, 2023, unvaccinated individuals constitute 22% of Venezuelans. This bias might be partially attributed to the high participation of healthcare workers and the low inclination of the unvaccinated group to participate in the survey, as observed in another KAP study conducted in Singapore [8]. Secondly, this study was based on an online survey with responses that were not verified, meaning the data are self-reported and dependent on the honesty of the participants. The results are likely influenced by social desirability bias and voluntary participation. Thirdly, the dissemination of online surveys via instant messaging, emails, and social networks could generate selection and self-selection bias, excluding participants without access to smart devices and/or the Internet connection, leading to underrepresentation or overrepresentation of certain groups. Therefore, the findings of this study should be interpreted with caution due to the specificity of the participants and are only applicable to populations with similar characteristics to those of this study. Recognizing these limitations, we propose strategic actions to enhance the quality of future research. These include diversifying recruitment channels beyond online platforms, collaborating with multiple research centers or institutions, stratifying the sample by Venezuelan states, and specifically encouraging the participation of non-vaccinated groups through community outreach, health facilities, or social media campaigns. Adhering to these recommendations could result in a larger and more diverse participant pool, thereby enhancing the validity and applicability of the results. However, there are also positive aspects of the study. These include the wide range of reasons related to the decision to vaccinate or not vaccinate against COVID-19, and a systematic form of data collection that avoided the inclusion of incomplete data or participants who did not meet the inclusion criteria. These findings may provide valuable information that could serve as a foundation for future research.

## Conclusions

This study describes an exploration of the population’s perception of COVID-19 vaccination in Venezuela, identifying sociodemographic variables related to KAP regarding the vaccine and COVID-19. The majority of participants demonstrated insufficient knowledge about the COVID-19 vaccine, particularly among the unvaccinated group. The scarcity of informational resources on disease transmission, vaccine efficacy, and vaccination schedules fostered vaccine hesitancy. To enhance equity in vaccination, public health campaigns in Venezuela should focus on implementing educational programs and increasing the availability of vaccination centers for the most vulnerable populations, characterized by low income, lower educational level, younger age, and residence in impoverished communities. Addressing these disparities in low- and middle-income countries is paramount in mitigating vaccine hesitancy.

### Electronic supplementary material

Below is the link to the electronic supplementary material.

Supplementary Material 1

Supplementary Material 2

Supplementary Material 3

Supplementary Material 4

Supplementary Material 5

## Data Availability

All data and materials in this article are included in the manuscript.

## Abbreviations

COVID-19: Coronavirus disease 2019
WHO: World Health Organization
KAP: Knowledge attitudes and practices
USA: United States of America
ENCOVI: National Survey of Living Conditions
SD: Standard deviation
IQR: Interquartile range
CI: Confidence interval
PAHO: Pan American Health Organization
