# Supplementary material for: Understanding the factors associated with COVID-19 vaccine hesitancy in Venezuela
Source: BMC Public Health. 2024 Apr 23;24:1117. doi: 10.1186/s12889-024-18598-4 (PMC11036563; doi:10.1186/s12889-024-18598-4)
Supplement: Supplementary file 1 — Supplementary Material 1 [file 12889_2024_18598_MOESM1_ESM.docx]

Survey on Knowledge, Attitudes, and Practices Related to the COVID-19 Vaccine in Venezuela

Dear Participant,

Venezuelan researchers at the Institute for Biomedical Research and Therapeutic Vaccines (VACTER) are conducting a survey on knowledge, attitudes, and practices related to the COVID-19 vaccine in Venezuela to understand how these three dimensions influence the decision to vaccinate or not in the Venezuelan population. Your participation is completely voluntary, anonymous, and conﬁdential.

You will not be asked at any time for your name or any other personal data.

We appreciate your participation in this study aimed at Venezuelans over 18 years of age residing in the country. Answering or not answering the questions will not be of any consequence to you. If you understand the purpose of this study and give your consent to participate, please select "Next".

* Indicates that the question is mandatory

1. V1. Where did you learn what you know about the COVID-19 vaccine? *

You may check more than one option

*Select all that apply.*

Facebook Twitter Instagram TikTok YouTube Radio Television

Newspapers

Educational talks

Scientific articles and/or magazines Advice from healthcare workers

Advice from family, friends, and/or neighbors

Internet (Google search)

Other:

1. V2. Do you wear a face mask properly (covering nose, mouth, and chin) at all times when you are in enclosed spaces and/or during mass events? *

## Mark one oval only

Always

Almost always

Frequently

Occasionally

Never

# V3. For you, should the COVID-19 vaccine be mandatory? *

## Mark one oval only

Totally agree

Agree

Neutral

Disagree
Totally disagree

# V4. Have you been vaccinated against COVID-19? *

## Mark one oval only

Yes *Skip to question 6*

No *Skip to question 5*

# NV1. Why have you not been vaccinated against COVID-19? *

You may check more than one option

*Select all that apply.*

I don't know where there is a vaccination center.

Vaccination centers operate at times similar to my work hours
 Vaccination centers are too far away from where I live

The lines to get vaccinated are too long

I expect to get immunity against COVID-19 by catching the virus

I do not trust the efficacy of the COVID-19 vaccines available in Venezuela
 I consider the COVID-19 vaccine to be unsafe for my health

I obtained the COVID-19 vaccination card without needing to be vaccinated
 COVID-19 does not represent a risk to my health or my family´s.

There is no COVID-19 virus.

Other:

*Skip to question 31*

# V5. Why did you get vaccinated against COVID-19? *

You may check more than one option

*Select all that apply.*

I was forced at work

To protect myself against COVID-19

To protect my family, friends, and/or neighbors against COVID-19
 It was a requirement to be able to travel out of the country.

My doctor recommended it

My family, friends, and/or neighbors recommended it to me

A famous person, inﬂuencer, and/or politician recommended it to me

A family member, friend, or neighbor took me to the vaccination center

Other:

# V6. What type of vaccine was administered? (1st dose) *

## Mark one oval only

Sputnik-V Sinopharm

Abdala (vaccine candidate) Soberana 02 (vaccine candidate) CanSino

AstraZeneca Sinovac/CoronaVac Johnson & Johnson Moderna

Pﬁzer

# V7. On what date was the 1st dose administered? *

*Example: January 7th 2019*

# V8. In which country was the 1st dose placed? *

## Mark one oval only

Venezuela

Other:

1. If the previous answer was "Venezuela", please mention in which state the 1st dose was placed

Mark one oval only

Amazonas Anzoátegui Apure Aragua Barinas Bolívar Carabobo Cojedes

Delta Amacuro Distrito Capital Falcón Guárico

Lara

La Guaira (Vargas) Mérida

Miranda Monagas Nueva Esparta Portuguesa Sucre

Táchira Trujillo Yaracuy Zulia

Dependencias Federales

1. V9. Did you get the 2nd dose of the COVID-19 vaccine? *

## Mark one oval only

Yes *Skip to question 12*

No *Skip to question 31*

# V10. What type of vaccine was administered? (2ª dose) *

## Mark one oval only

Sputnik-V Sinopharm

Abdala (vaccine candidate) Soberana 02 (vaccine candidate) CanSino

AstraZeneca Sinovac/CoronaVac Johnson & Johnson Moderna

Pﬁzer

# V11. On what date was the 2nd dose administered? *

*Example: January 7th 2019*

# V12. In which country was the 2nd dose placed? *

## Mark one oval only

Venezuela

Other:

1. If the previous answer was "Venezuela", please mention in which state the 2nd dose was placed

## Mark one oval only

Amazonas Anzoátegui Apure Aragua Barinas Bolívar Carabobo Cojedes

Delta Amacuro Distrito Capital Falcón Guárico

Lara

La Guaira (Vargas) Mérida

Miranda Monagas Nueva Esparta Portuguesa Sucre

Táchira Trujillo Yaracuy Zulia

Dependencias Federales

1. V13. Did you receive the 3rd dose of COVID-19 vaccine?? *

Mark one oval only

Yes

No *Skip to question 31*

# V14. What type of vaccine was administered? (3rd dose) *

## Mark one oval only

Sputnik-V Sinopharm

Abdala (vaccine candidate) Soberana 02 (vaccine candidate) CanSino

AstraZeneca Sinovac/CoronaVac Johnson & Johnson Moderna

Pﬁzer

# V15. On what date was the 3rd dose administered? *

*Example: January 7th 2019*

1. V16. In which country was the 3rd dose placed? *

Mark one oval only

Venezuela

Other:

1. If the previous answer was "Venezuela", mention in which state the 3rd dose was given

## Mark one oval only

Amazonas Anzoátegui Apure Aragua Barinas Bolívar Carabobo Cojedes

Delta Amacuro Distrito Capital Falcón Guárico

Lara

La Guaira (Vargas) Mérida

Miranda Monagas Nueva Esparta Portuguesa Sucre

Táchira Trujillo Yaracuy Zulia

Dependencias Federales

1. V17. Did you get the 4th dose of the COVID-19 vaccine? *

## Mark one oval only

Yes

No *Skip to question 31*

# V18. What type of vaccine was administered? (4th dose) *

## Mark one oval only

Sputnik-V Sinopharm

Abdala (vaccine candidate) Soberana 02 (vaccine candidate) CanSino

AstraZeneca Sinovac/CoronaVac Johnson & Johnson Moderna

Pﬁzer

# V19. On what date was the 4th dose given? *

*Example: January 7th 2019*

# V20. In which country was the 4th dose placed? *

## Mark one oval only

Venezuela

Other:

1. If the previous answer was "Venezuela", mention in which state the 4th dose was given

Mark one oval only

Amazonas Anzoátegui Apure Aragua Barinas Bolívar Carabobo Cojedes

Delta Amacuro Distrito Capital Falcón Guárico

Lara

La Guaira (Vargas) Mérida

Miranda Monagas Nueva Esparta Portuguesa Sucre

Táchira Trujillo Yaracuy Zulia

Dependencias Federales

1. V21. Did you receive the 5th dose of COVID-19 vaccine?

## Mark one oval only

Yes

No *Skip to question 31*

# V22. What type of vaccine was administered? (5th dose) *

## Mark one oval only

Sputnik-V Sinopharm

Abdala (vaccine candidate) Soberana 02 (vaccine candidate) CanSino

AstraZeneca Sinovac/CoronaVac Johnson & Johnson Moderna

Pﬁzer

# V23. On what date was the 5th dose administered? *

*Example: January 7th 2019*

# V24. In which country was the 5th dose placed? *

## Mark one oval only

Venezuela

Other:

1. If the previous answer was "Venezuela", mention in which state the 5th dose was given.

Mark one oval only

Amazonas Anzoátegui Apure Aragua Barinas Bolívar Carabobo Cojedes

Delta Amacuro Distrito Capital Falcón Guárico

Lara

La Guaira (Vargas) Mérida

Miranda Monagas Nueva Esparta Portuguesa Sucre

Táchira Trujillo Yaracuy Zulia

Dependencias Federales

1. C1. COVID-19 vaccine decreases risk of developing severe COVID-19 and dying

*

## Mark one oval only

True
 False

I do not know

# C2. COVID-19 vaccine helps to protect the community against the virus *

## Mark one oval only

True
 False

I do not know

# C3. It is possible to become ill with COVID-19 because you have been vaccinated against COVID-19 *

## Mark one oval only

True
 False

I do not know

1. C4. The COVID-19 vaccine may produce minor side effects, such as fatigue, fever and malaise. *

## Mark one oval only

True

False

I do not know

# C5. Some COVID-19 vaccines are more effective than others. *

## Mark one oval only

True

False

I do not know

# C6. Available vaccines against COVID-19 are less efﬁcient against newer variants of the virus (e.g., Omicron). *

## Mark one oval only

True

False

I do not know

# C7. Getting vaccinated against COVID-19 has more risks than beneﬁts. *

## Mark one oval only

True

False

I do not know

1. C8. Booster doses of the COVID-19 vaccine increase protection against the virus. *

## Mark one oval only

True

False

I do not know

# C9. It is recommended that people with risk factors for developing severe COVID-19, such as hypertension and diabetes, be vaccinated against COVID-19. *

## Mark one oval only

True

False

I do not know

# C10. Natural immunity (catching the virus) can be boosted with the COVID-19 vaccine. *

## Mark one oval only

True

False

I do not know

# C11. From 6 months of age, all persons can receive the COVID-19 vaccine. *

## Mark one oval only

True

False

I do not know

# C12. Pregnant women can be vaccinated against COVID-19*

## Mark one oval only

True

False

I do not know

# A1. The COVID-19 vaccine is safe *

## Mark one oval only

Totally agree

Agree

Neutral

Disagree
Totally disagree

# A2. The COVID-19 vaccine may help to stop the pandemic *

## Mark one oval only

Totally agree
Agree

Neutral

Disagree
Totally disagree

# A3. SARS-CoV-2 exists (virus causing COVID-19) *

## Mark one oval only

Totally agree

Agree

Neutral

Disagree
Totally disagree

# A4. Despite the accelerated development of the COVID-19 vaccine, I am confident of its efﬁciency. *

## Mark one oval only

Totally agree
Agree

Neutral

Disagree
Totally disagree

# A5. I trust in the transparency of pharmaceutical companies on the safety of COVID-19 vaccines. *

## Mark one oval only

Totally agree
Agree

Neutral

Disagree
Totally disagree

# A6. If beneficial, I would have a booster dose of the COVID-19 vaccine annually *

## Mark one oval only

Totally agree
Agree

Neutral

Disagree
Totally disagree

# A7. I am willing to be vaccinated against COVID-19 even if I have to pay for it. *

## Mark one oval only

Totally agree
Agree

Neutral

Disagree
Totally disagree

# A8. COVID-19 vaccines available in Venezuela are efﬁcient *

## Mark one oval only

Totally agree
Agree

Neutral

Disagree
Totally disagree

# A9. Vaccination against COVID-19 is beneficial although the number of cases is currently low in Venezuela *

## Mark one oval only

Totally agree
Agree

Neutral

Disagree
Totally disagree

# A10. I rely on the protocols used at the COVID-19 vaccination sites, such as biosecurity, hygiene, and organizational measures. *

## Mark one oval only

Totally agree
Agree

Neutral

Disagree
Totally disagree

# P1. I am looking for up-to-date and trustworthy information on the COVID-19 vaccine*

## Mark one oval only

Always
Almost always
Frequently

Occasionally
Never

# P2. I recommend that my family, friends, and/or neighbors to get vaccinated against COVID-19 *

## Mark one oval only

Always
Almost always
Frequently

Occasionally
Never

# P3. I am disseminating information on vaccination campaigns against COVID-19 *

## Mark one oval only

Always
Almost always
Frequently

Occasionally
Never

1. P4. I combat misinformation (unveriﬁed/untrustworthy information) regarding the COVID-19 vaccine *

## Mark one oval only

Always
Almost always
Frequently

Occasionally
Never

# P5. I get vaccinated annually against influenza/flu *

## Mark one oval only

Always
Almost always
Frequently

Occasionally
Never

# D1. What is your gender? *

## Mark one oval only

Male
Female
Other:

# D2. How old are you? *

1. D3. What is your marital status? *

## Mark one oval only

Single

Cohabitant (free union)
Married

Divorced
Widowed

# D4. What is your highest level of education attained? *

## Mark one oval only

None
Elementary Secondary

Technician Bachelors
University

# D5. What is your occupation? *

## Mark one oval only

I work full-time or part-time (employed)
 I work in the health care field (health personnel)
I am self-employed (independent)

I am unemployed
 I am a student
I am retired

# D6. Do you currently have any of the following diseases? *

You may check more than one option

*Select all that apply.*

Hypertension

Chronic obstructive pulmonary disease (COPD)
Diabetes

Human immunodeﬁciency virus (HIV)
Cancer

Chronic kidney disease (CKD)
Asthma

Hypothyroidism
Obesity
Heart disease

Autoimmune or rheumatologic disease
None

Other:

1. D7. What religion do you practice? *

*Mark one oval only*

Catholic
Mormon
Evangelical

Jehovah's Witness
Muslim
Agnostic
Shriner

Buddhist
 Hindu
Jewish
Atheist
Other:

1. D8. In which state of Venezuela do you live? *

*Mark one oval only*

Amazonas Anzoátegui Apure Aragua Barinas Bolívar Carabobo Cojedes

Delta Amacuro Distrito Capital Falcón Guárico

Lara

La Guaira (Vargas) Mérida

Miranda Monagas Nueva Esparta Portuguesa Sucre

Táchira Trujillo Yaracuy Zulia

Dependencias Federales

# D9. In which municipality of Venezuela do you live? *

1. D10. What is your area of residence? *

## Mark one oval only

City or urban area (living and working area with high population)

Village or suburban area (single-family homes that are close together with a higher population than rural but lower than urban)

Village or rural area (open and dispersed area with low population)

# D11. How much is your monthly income? *

## Mark one oval only

Less than $100
Between $101 and $200
 Between $201 and $300
 More than $300

This content has not been created or approved by Google.

[Formularies](https://www.google.com/forms/about/?utm_source=product&utm_medium=forms_logo&utm_campaign=forms)
