# Supplementary material for: Understanding the factors associated with COVID-19 vaccine hesitancy in Venezuela
Source: BMC Public Health. 2024 Apr 23;24:1117. doi: 10.1186/s12889-024-18598-4 (PMC11036563; doi:10.1186/s12889-024-18598-4)
Supplement: Supplementary file 2 — Supplementary Material 2 [file 12889_2024_18598_MOESM2_ESM.docx]

**Supplementary Data 2.** Levels of difficulty and discrimination of the “knowledge” dimension in the pilot test

| **Items** | **Difficulty** | **Discrimination** |
| --- | --- | --- |
| Item 1. COVID-19 vaccine decreases the risk of developing severe COVID-19 and dying | 0.858 | 0.469 |
| Item 2. COVID-19 vaccine helps protect the community against the virus | 0.825 | 0.406 |
| Item 3. It is possible to become sick with COVID-19 because of vaccination | 0.317 | 0.531 |
| Item 4. The COVID-19 vaccine may cause minor side effects, such as fatigue, fever, and malaise | 0.875 | 0.188 |
| Item 5. Some COVID-19 vaccines are more effective than others | 0.617 | 0.532 |
| Item 6. Available COVID-19 vaccines are less effective against newer variants of the virus (e.g., Omicron) | 0.258 | 0.406 |
| Item 7. Vaccination against COVID-19 has more risks than benefits | 0.717 | 0.625 |
| Item 8. Booster doses of COVID-19 vaccine increase protection against the virus | 0.708 | 0.75 |
| Item 9. It is recommended that people with risk factors for developing severe COVID-19, such as hypertension and diabetes, be vaccinated against COVID-19 | 0.792 | 0.562 |
| Item 10. Natural immunity (catching the virus) may be boosted with the COVID-19 vaccine | 0.592 | 0.781 |
| Item 11. Starting at six months of age, all persons may receive the COVID-19 vaccine, such as Pfizer or Moderna | 0.183 | 0.407 |
| Item 12. Pregnant women may be vaccinated against COVID-19 | 0.358 | 0.906 |
