# Supplementary material for: Understanding the factors associated with COVID-19 vaccine hesitancy in Venezuela
Source: BMC Public Health. 2024 Apr 23;24:1117. doi: 10.1186/s12889-024-18598-4 (PMC11036563; doi:10.1186/s12889-024-18598-4)
Supplement: Supplementary file 3 — Supplementary Material 3 [file 12889_2024_18598_MOESM3_ESM.docx]

**Supplementary Data 3.** Reasons related to the decision of whether or not to be vaccinated against COVID-19 of 1,930 Venezuelan participants

| **Other features about the COVID-19 vaccination** |  |
| --- | --- |
| Why have you not gotten the COVID-19 vaccine? | ***n* = 128** |
| I consider that the COVID-19 vaccine is not safe for my health | 75 (58.6) |
| I do not trust the efficacy of the COVID-19 vaccines available in Venezuela | 65 (50.8) |
| I expect to obtain immunity against COVID-19 by being infected with the virus | 24 (18.8) |
| COVID-19 does not pose a health risk to me or my family | 8 (6.3) |
| Vaccination centers operate on similar schedules to my work schedule | 5 (3.9) |
| I got my COVID-19 vaccination card without the need for vaccination | 5 (3.9) |
| I don’t know where there is a vaccination center | 4 (3.1) |
| The lines to get vaccinated are very long | 4 (3.1) |
| There is no such thing as COVID-19 virus | 4 (3.1) |
| The vaccination centers are far away from where I live | 1 (0.8) |
| Other | 14 (0.7) |
| Why did you get the COVID-19 vaccine? | ***n* = 1,802** |
| To protect myself against COVID-19 | 1,468 (81.5) |
| To protect my family, friends and/or neighbors against COVID-19 | 1,304 (72.4) |
| My doctor recommended it to me | 275 (15.3) |
| It was a requirement to travel out of the country | 246 (13.7) |
| My family, friends and/or neighbors recommended it to me | 209 (11.6) |
| I was forced to do it at work | 82 (4.6) |
| A family member, friend or neighbor took me to the vaccination center | 67 (3.7) |
| A celebrity, influencer, and/or politician recommended it | 17 (0.9) |
| Other | 43 (2.4) |
