# Supplementary material for: Understanding the factors associated with COVID-19 vaccine hesitancy in Venezuela
Source: BMC Public Health. 2024 Apr 23;24:1117. doi: 10.1186/s12889-024-18598-4 (PMC11036563; doi:10.1186/s12889-024-18598-4)
Supplement: Supplementary file 4 — Supplementary Material 4 [file 12889_2024_18598_MOESM4_ESM.docx]

**Supplementary Data 4.** Attitudes towards the COVID-19 vaccine among vaccinated and unvaccinated participants

| **Attitudes** | **Total (*n* = 1,930; 100%)** | **Vaccinated (*n* = 1,802; 93.4%)** | **Unvaccinated (*n* = 128; 6.6%)** | ***P*-value** |
| --- | --- | --- | --- | --- |
| Attitudes, mean (SD), points | 39 (8) | 40 (7) | 24 (7) | <0.001^*^ |
| Attitudes, *n* (%) |  |  |  | <0.001^†‡^ |
| Negative (≤29 points) | 229 (11.9) | 131 (7.3) | 98 (76.6) |  |
| Indifferent (30–39 points) | 638 (33.1) | 609 (33.8) | 29 (22.7) |  |
| Positive (≥40 points) | 1,063 (55.1) | 1,062 (58.9) | 1 (0.8) |  |
| COVID-19 vaccine is safe, *n* (%) |  |  |  |  |
| Strongly agree | 696 (36.1) | 694 (38.5) | 2 (1.6) | <0.001^†§^ |
| Agree | 679 (35.2) | 668 (37.1) | 11 (8.6) | <0.001^†§^ |
| Neutral | 396 (20.5) | 358 (19.9) | 38 (29.7) | <0.001^†§^ |
| Disagree | 105 (5.4) | 63 (3.5) | 42 (32.8) | <0.001^†§^ |
| Strongly disagree | 54 (2.8) | 19 (1.1) | 35 (27.3) | <0.001^†§^ |
| COVID-19 vaccine may help stop the pandemic, *n* (%) |  |  |  |  |
| Strongly agree | 848 (43.9) | 844 (46.8) | 4 (3.1) | <0.001^†§^ |
| Agree | 618 (32) | 604 (33.5) | 14 (10.9) | <0.001^†§^ |
| Neutral | 307 (15.9) | 269 (14.9) | 38 (29.7) | <0.001^†§^ |
| Disagree | 106 (5.5) | 62 (3.4) | 44 (34.4) | <0.001^†§^ |
| Strongly disagree | 51 (2.6) | 23 (1.3) | 28 (21.9) | <0.001^†§^ |
| SARS-CoV-2 exists (virus causing COVID-19), *n* (%) |  |  |  |  |
| Strongly agree | 1,250 (64.8) | 1,214 (67.4) | 36 (28.1) | <0.001^†§^ |
| Agree | 449 (23.3) | 396 (22) | 53 (41.4) | <0.001^†§^ |
| Neutral | 208 (10.8) | 176 (9.8) | 32 (25) | <0.001^†§^ |
| Disagree | 18 (0.9) | 14 (0.8) | 4 (3.1) | 0.008^†§^ |
| Strongly disagree | 5 (0.3) | 2 (0.1) | 3 (2.3) | <0.001^†§^ |
| Despite the accelerated development of the COVID-19 vaccine, I am confident in its efficacy, *n* (%) |  |  |  |  |
| Strongly agree | 700 (36.3) | 697 (38.7) | 3 (2.3) | <0.001^†§^ |
| Agree | 733 (38) | 724 (40.2) | 9 (7) | <0.001^†§^ |
| Neutral | 295 (15.3) | 271 (15) | 24 (18.8) | 0.259^†§^ |
| Disagree | 130 (6.7) | 84 (4.7) | 46 (35.9) | <0.001^†§^ |
| Strongly disagree | 72 (3.7) | 26 (1.4) | 46 (35.9) | <0.001^†§^ |
| I trust the transparency of pharmaceutical companies about the safety of COVID-19 vaccines, *n* (%) |  |  |  |  |
| Strongly agree | 432 (22.4) | 431 (23.9) | 1 (0.8) | <0.001^†§^ |
| Agree | 583 (30.2) | 579 (32.1) | 4 (3.1) | <0.001^†§^ |
| Neutral | 623 (32.3) | 591 (32.8) | 32 (25) | 0.068^†§^ |
| Disagree | 170 (8.8) | 134 (7.4) | 36 (28.1) | <0.001^†§^ |
| Strongly disagree | 122 (6.3) | 67 (3.7) | 55 (43) | <0.001^†§^ |
| If beneficial, I would get an annual booster dose of the COVID-19 vaccine, *n* (%) |  |  |  |  |
| Strongly agree | 863 (44.7) | 856 (47.5) | 7 (5.5) | <0.001^†§^ |
| Agree | 561 (29.1) | 537 (29.8) | 24 (18.8) | 0.008^†§^ |
| Neutral | 261 (13.5) | 233 (12.9) | 28 (21.9) | 0.004^†§^ |
| Disagree | 139 (7.2) | 109 (6) | 30 (23.4) | <0.001^†§^ |
| Strongly disagree | 106 (5.5) | 67 (3.7) | 39 (30.5) | <0.001^†§^ |
| I am willing to get vaccinated against COVID-19 even if I have to pay for it, *n* (%) |  |  |  |  |
| Strongly agree | 491 (25.4) | 486 (27) | 5 (3.9) | <0.001^†§^ |
| Agree | 485 (25.1) | 482 (26.7) | 3 (2.3) | <0.001^†§^ |
| Neutral | 421 (21.8) | 403 (22.4) | 18 (14.1) | 0.028^†§^ |
| Disagree | 331 (17.2) | 287 (15.9) | 4 (34.4) | <0.001^†§^ |
| Strongly disagree | 202 (10.5) | 144 (8) | 58 (45.3) | <0.001^†§^ |
| COVID-19 vaccines available in Venezuela are effective, *n* (%) |  |  |  |  |
| Strongly agree | 459 (23.8) | 457 (25.4) | 2 (1.6) | <0.001^†§^ |
| Agree | 834 (43.2) | 825 (45.8) | 9 (7) | <0.001^†§^ |
| Neutral | 487 (25.2) | 442 (24.5) | 45 (35.2) | 0.008^†§^ |
| Disagree | 95 (4.9) | 60 (3.3) | 35 (27.3) | <0.001^†§^ |
| Strongly disagree | 55 (2.8) | 18 (1) | 37 (28.9) | <0.001^†§^ |
| Vaccination against COVID-19 is beneficial, although the number of cases is currently low in Venezuela, *n* (%) |  |  |  |  |
| Strongly agree | 843 (43.7) | 841 (46.7) | 2 (1.6) | <0.001^†§^ |
| Agree | 660 (34.2) | 648 (36) | 12 (9.4) | <0.001^†§^ |
| Neutral | 289 (15) | 245 (13.6) | 44 (34.4) | 0.008^†§^ |
| Disagree | 84 (4.4) | 48 (2.7) | 36 (28.1) | <0.001^†§^ |
| Strongly disagree | 54 (2.8) | 20 (1.1) | 34 (26.6) | <0.001^†§^ |
| I trust the protocols used at COVID-19 vaccination sites, such as biosecurity, hygiene and organizational measures, *n* (%) |  |  |  |  |
| Strongly agree | 628 (32.5) | 617 (34.2) | 11 (8.6) | <0.001^†§^ |
| Agree | 688 (35.6) | 670 (37.2) | 18 (14.1) | <0.001^†§^ |
| Neutral | 436 (22.6) | 394 (21.9) | 42 (32.8) | 0.004^†§^ |
| Disagree | 122 (6.3) | 96 (5.3) | 26 (20.3) | <0.001^†§^ |
| Strongly disagree | 56 (2.9) | 25 (1.4) | 31 (24.2) | <0.001^†§^ |

^*^Student’s *t*-test for independent samples; ^†^Pearson’s chi-square test; ^‡^Significant only for Negative (*p* < 0.001) and for Positive (*p* < 0.001) for a value of α ≤ 0.008 by Bonferroni correction; ^§^Significant only for a value of α ≤ 0.005 by Bonferroni correction.
