# Supplementary material for: Understanding the factors associated with COVID-19 vaccine hesitancy in Venezuela
Source: BMC Public Health. 2024 Apr 23;24:1117. doi: 10.1186/s12889-024-18598-4 (PMC11036563; doi:10.1186/s12889-024-18598-4)
Supplement: Supplementary file 5 — Supplementary Material 5 [file 12889_2024_18598_MOESM5_ESM.docx]

**Supplementary Data 5.** Practices related to the COVID-19 vaccine among vaccinated and unvaccinated participants

| **Practices** | **Total (*n* = 1,930; 100%)** | **Vaccinated (*n* = 1,802; 93.4%)** | **Unvaccinated (*n* = 128; 6.6%)** | ***P*-value** |
| --- | --- | --- | --- | --- |
| Practices, mean (SD), points | 16 (6) | 16 (5) | 10 (3) | <0.001^*^ |
| Practices, *n* (%) |  |  |  | <0.001^†‡^ |
| Inappropriate (≤14 points) | 799 (41.4) | 682 (37.8) | 117 (91.4) |  |
| Indifferent (15–19 points) | 504 (26.1) | 493 (27.4) | 11 (8.6) |  |
| Appropriate (≥20 points) | 627 (32.5) | 627 (34.8) | 0 (0) |  |
| I seek up-to-date and reliable information about the COVID-19 vaccine, *n* (%) |  |  |  |  |
| Always | 587 (30.4) | 559 (31) | 28 (21.9) | 0.030^†§^ |
| Almost always | 390 (20.2) | 369 (20.5) | 21 (16.4) | 0.060^†§^ |
| Often | 318 (16.5) | 302 (16.8) | 16 (12.5) | <0.001^†§^ |
| Occasionally | 484 (25.1) | 443 (24.6) | 41 (32) | 0.268^†§^ |
| Never | 151 (7.8) | 129 (7.2) | 22 (17.2) | 0.209^†§^ |
| I recommend to my family, friends and/or neighbors to get vaccinated against COVID-19, *n* (%) |  |  |  |  |
| Always | 1039 (53.8) | 1038 (57.6) | 1 (0.8) | <0.001^†§^ |
| Almost always | 228 (11.8) | 224 (12.4) | 4 (3.1) | 0.647^†§^ |
| Often | 177 (9.2) | 173 (9.6) | 4 (3.1) | <0.001^†§^ |
| Occasionally | 203 (10.5) | 188 (10.4) | 15 (11.7) | 0.002^†§^ |
| Never | 283 (14.7) | 179 (9.9) | 104 (81.3) | 0.014^†§^ |
| I disseminate information about COVID-19 vaccination campaigns, *n* (%) |  |  |  |  |
| Always | 514 (26.6) | 512 (28.4) | 2 (1.6) | <0.001^†§^ |
| Almost always | 267 (13.8) | 263 (14.6) | 4 (3.1) | <0.001^†§^ |
| Often | 255 (13.2) | 252 (14) | 3 (2.3) | <0.001^†§^ |
| Occasionally | 397 (20.6) | 389 (21.6) | 8 (6.3) | <0.001^†§^ |
| Never | 497 (25.8) | 386 (21.4) | 111 (86.7) | <0.001^†§^ |
| I combat misinformation (unverified/unreliable information) regarding the COVID-19 vaccine, *n* (%) |  |  |  |  |
| Always | 701 (36.3) | 681 (37.8) | 20 (15.6) | <0.001^†§^ |
| Almost always | 350 (18.1) | 341 (18.9) | 9 (7) | 0.059^†§^ |
| Often | 287 (14.9) | 271 (15) | 16 (12.5) | <0.001^†§^ |
| Occasionally | 308 (16) | 280 (15.5) | 28 (21.9) | <0.001^†§^ |
| Never | 284 (14.7) | 229 (12.7) | 55 (43) | 0.435^†§^ |
| I get vaccinated annually against influenza/flu, *n* (%) |  |  |  |  |
| Always | 259 (13.4) | 253 (14) | 6 (4.7) | 0.003^†§^ |
| Almost always | 267 (13.8) | 260 (14.4) | 7 (5.5) | 0.034^†§^ |
| Often | 183 (9.5) | 171 (9.5) | 12 (9.4) | <0.001^†§^ |
| Occasionally | 466 (24.1) | 445 (24.7) | 21 (16.4) | 0.005^†§^ |
| Never | 755 (39.1) | 673 (37.3) | 82 (64.1) | 0.966^†§^ |

^*^Student’s *t*-test for independent samples; ^†^Pearson’s chi-square test; ^‡^Significant only for Inappropriate (*p* < 0.001) and for Appropriate (*p* < 0.001) for a value of α ≤ 0.008 by Bonferroni correction; ^§^Significant only for a value of α ≤ 0.005 by Bonferroni correction.
